# DIFFERENCES IN HOSPITALIZATIONS, ER ADMISSIONS, AND OUTPATIENT VISITS FOR MEXICAN-AMERICANS AGE 75 AND OLDER

**DOI:** 10.1093/geroni/igz038.2082

**Published:** 2019-11-08

**Authors:** Brian Downer, Soham Al Snih, Lin-Na Chou, Yong-Fang Kuo, Kyriakos Markides, Kenneth Ottenbacher

**Affiliations:** 1 University of Texas Medical Branch, Galveston, TX, Galveston, Texas, United States; 2 University of Texas Medical Branch, Galveston, Texas, United States

## Abstract

Few studies have investigated the healthcare utilization of Mexican-American Medicare beneficiaries. We used data from 1,196 Hispanic-EPESE participants aged >75 years that has been linked with Medicare claims to describe the healthcare utilization of older Mexican-Americans and determine common reasons for hospitalizations. Participants were followed for two-years (eight-quarters). We estimated the probability of >1 hospitalization, emergency room (ER) admissions, and outpatient visits per quarter. The percentage of participants who had >1 hospitalizations, ER admissions, and outpatient visits for each quarter ranged from 10.6%-13.2%, 14.6%-19.5%, and 77.2%-80.5%, respectively. Twenty-three percent of hospitalizations were for circulatory conditions and 17% were for respiratory conditions. Older age (OR=1.26) and Spanish language (OR=1.51) were associated with hospitalizations. Women had higher odds than men to have an outpatient visit (OR=1.61). Greater education was associated with ER admissions (OR=0.72). Continued research is needed to identify social determinants and health characteristics associated with healthcare utilization among older Mexican-Americans.

